# Are prenatal mercury levels associated with subsequent blood pressure in childhood and adolescence? The Avon prebirth cohort study

**DOI:** 10.1136/bmjopen-2016-012425

**Published:** 2016-10-14

**Authors:** Steve Gregory, Yasmin Iles-Caven, Joseph R Hibbeln, Caroline M Taylor, Jean Golding

**Affiliations:** 1 Centre for Child and Adolescent Health, School of Social and Community Medicine, University of Bristol, Bristol, UK; 2 Department of Nutritional Neurosciences, LMBB, National Institute on Alcohol Abuse and Alcoholism, National Institutes of Health, Bethesda, Maryland, USA

**Keywords:** PUBLIC HEALTH, TOXICOLOGY, ALSPAC, Cardiovascular system, Prenatal mercury, Selenium

## Abstract

**Objectives:**

There have been conflicting data suggesting that prenatal mercury exposure is associated with adverse cardiovascular measures in children. We therefore analysed a large prospective population study to investigate whether prenatal mercury exposure might influence offspring blood pressure (BP) and heart rate adversely.

**Design:**

Prospective birth cohort.

**Setting:**

The Avon Longitudinal Study of Parents and Children (ALSPAC).

**Participants:**

Maternal whole blood collected in the first half of pregnancy was assayed for mercury and selenium. The offspring were followed throughout childhood and adolescence.

**Outcome measures:**

Offspring resting BP and heart rates measured under standard conditions on six occasions between ages 7 and 17 years (numbers analysed: 1754 at 7 years to 1102 at 17).

**Results:**

Statistical analyses took account of various factors present in pregnancy, including family adversity, maternal age, parity, smoking and alcohol intake. Unadjusted and adjusted regression analyses assessed the relationship between maternal prenatal mercury levels and offspring resting systolic and diastolic BP, and heart rates. A final set of analyses took account of selenium. Each analysis was carried out for all offspring, those whose mothers had, and those that had not, consumed fish during pregnancy. Further analysis for all offspring ascertained whether there were significant interaction effects between the sexes. There was little evidence to suggest that prenatal mercury exposure resulted in a clinically important increase in offspring BP in the whole group, since no effect size for an increase of 1 SD of blood mercury level was >0.3 mm Hg. Only 1 association was significant at p<0.05 and therefore likely due to chance.

**Conclusions:**

This study reveals no evidence to support the hypothesis that prenatal mercury exposure has adverse long-term effects on offspring BP or heart rates during childhood or adolescence.

**Strengths and limitations of this study**

- The benefits included the large numbers relative to other studies, its prospective nature and the standardised conditions under which the blood pressures were taken.
- The study was able to compare the effects on the children whose mothers ate fish with those who did not.
- Like all cohort studies, there was a loss to follow-up, which differentially concerned the more socioeconomically disadvantaged. It is possible that this may have biased the results.
- The study was concerned with levels of blood mercury that were similar to those of Western Europe and the USA, but cannot be extrapolated to areas where sea mammals are eaten such as the Faroe Islands, where the levels of mercury are much higher.

## Introduction

Although there is agreement that consumption of fish is beneficial for the heart,^1^ this is thought to be due to the benefits of the omega-3 fatty acids in fish, especially oily fish. However, there is still concern over the mercury content of fish, as there is some evidence of increased levels of mercury exposure being associated with increases in blood pressure (BP).^2^ Problems with many of the published studies concern the small numbers involved, cross-sectional analyses and a failure to assess the inter-relationship with omega-3 intake. Consequently, it is not possible from these studies to interpret whether there is a consistent causal sequence.

Two major longitudinal studies have addressed the question as to whether there is a link between mercury measured in toenails and subsequent coronary heart disease (CHD); the authors collected toenails from tens of thousands of individuals and compared the mercury levels of those who developed CHD with the levels in controls—neither study showed an association between mercury level and either CHD or stroke.^3^ ^4^ However, these studies did not take account of the beneficial influence of fish intake on the heart. That this has an advantage is illustrated by the findings of Vupputuri *et al*^5^ who analysed cross-sectional information from the American NHANES observational study. Initially, there was no association between mercury and BP when the data were analysed together. However, when stratified by consumption of fish, there was a significant positive association between mercury and BP, but only in the non-fish-eaters.

These studies were all of adults. Animal studies have indicated that deficiency in omega-3 fatty acids in the pregnant rat is associated with higher BP in the offspring.^6^ In humans, a randomised controlled trial has shown that maternal prenatal diet can influence offspring BP,^7^ but, to the best of our knowledge, only three studies have assessed effects of prenatal mercury exposure on offspring BP.^8–10^ Given that, according to the Developmental Origins of Health and Disease hypothesis,^11^ fetal life is important for programming the development of cardiovascular disease, in this paper, our aim is to assess whether maternal prenatal blood mercury is associated with childhood and/or adolescent BP, and whether any relationship differs between offspring whose mothers ate fish in pregnancy and those who did not. We use the prospectively studied Avon Longitudinal Study of Parents and Children (ALSPAC), which has the advantage of larger numbers than previously studied, and measures of BP throughout childhood and adolescence.

## Methods and materials

### The ALSPAC cohort

The ALSPAC study aimed to enrol all pregnant women residing in Avon (a geographically defined area that includes the city of Bristol, surrounding smaller urban towns, and rural areas about 120 miles west of London, UK) with an expected delivery date between 1 April 1991 and 31 December 1992. The study enrolled 14 541 pregnant women, estimated as about 80% of those eligible. Its stated aims were to evaluate genetic and environmental influences on health and development, including environmental factors measured prospectively during pregnancy.^12^ ^13^

### Prenatal trace metal exposures

Blood samples deliberately collected in acid-washed containers for determination of trace metals were obtained from 4484 women residing in two of the three Health Authority areas of the recruitment region. Samples were collected by midwives as early as possible in pregnancy. The sociodemographic characteristics of the women who donated samples were comparable to those of the rest of the ALSPAC study population apart from including a slight excess of older and more educated mothers.^14^

Gestational age at sample collection (known for 4472 mothers (99.7%)) had a median value of 11 weeks and mode of 10 weeks. The IQR was 9–13 weeks; 93% of the samples were collected at <18 weeks gestation. Samples were stored for 0–4 days at 4°C at the collection site before being sent to the central Bristol laboratory. Samples were transported at room temperature for up to 3 hours, and stored at 4°C as whole blood in the original collection tubes for 18–19.5 years before analysis.^15^

Analyses of the blood samples for whole blood mercury and selenium were carried out by the laboratory of Dr Robert Jones at the Centers for Disease Control and Prevention (CDC) (CDC method 3009.1; unpublished information). Clotted whole blood was digested to remove all clots, before being analysed using inductively coupled plasma dynamic reaction cell mass spectrometry (ICP-DRC-MS).^16–19^

The entire amount of clotted whole blood was transferred to a digestion tube using concentrated nitric acid with the volume estimated from the weight.^20^ The blood sample was heated in a microwave oven at a controlled temperature and time during which the organic matrix of the blood was digested removing the clots. ICP-DRC-MS internal standards (iridium and tellurium) were added at a constant concentration to all blanks, calibrators and samples (at the time of 1:9 dilution of digestate) to facilitate correction for instrument noise and drift. The standard additions method of calibration was used to optimise the analytical sensitivity of the method for the whole blood samples. A recovery spike was included in each analytical run for calibration verification and as a blind quality control (QC) sample. The ICP-DRC-MS was operated in the DRC mode using oxygen when analysing for mercury and selenium. QC materials as well as inhouse QC samples with control limits unknown to the analysts were used for daily QC.

There were no samples with selenium levels below the limit of detection (LOD) for selenium. The blood selenium levels ranged from 17.0 to 324.1 µg/L, with 5th, 10th, 50th, 90th and 95th centiles of 81.4, 86.7, 108.0, 139.0 and 152.5 µg/L. The LOD for mercury was 0.24 µg/L; three samples were below this level and were ascribed a value of 0.7 times the LOD (since the frequency distribution of mercury had evidence of a lower tail, a factor >0.5 was deemed appropriate to reflect the likelihood that more of these three results would be closer to the LOD than zero). The maternal blood mercury levels ranged from 0.17 to 12.76 µg/L, with 5th, 10th, 50th, 90th and 95th centiles of 0.81, 0.99, 1.86, 3.33 and 4.02 µg/L, respectively.

### Maternal dietary information

A questionnaire sent to the mother at 32 weeks gestation included a food frequency questionnaire comprising 103 food and drink items, including 3 items related to seafood: white fish, oily fish and shellfish. The participants were given guidelines to classify the three types using seafood categories that are most prevalent in the UK. Thus, oily fish was described as including ‘salmon, mackerel, sardines, trout, herring, pilchards, tuna, etc’; white fish as including ‘cod, haddock, plaice, fish fingers etc’ and shellfish as including ‘prawns, mussels, cockles, crab etc’. The woman was asked how frequently she was currently eating each of these groups, with options: ‘not at all; about once in 2 weeks; 1–3 times a week; 4–7 times a week; more than once a day’.^21^ In the analyses used here, we defined fish-eaters as those women reporting eating any white or oily fish.

### Outcome measures

Resting BP and heart rates were measured at 7, 9, 11, 13, 15 and 17 years using a Dinamap 9301 Vital Signs Monitor at a specially designed clinic. The 1994 OPCS Dinamap Calibration Study^22^ indicated that this device was highly reliable with repeat measures yielding correlation coefficients of 0.88 for systolic BP and 0.83 for diastolic BP. For ages up to 13, these measurements were taken in a relaxed atmosphere, and were followed by a discussion concerning whether to have blood taken and, if so, having an anaesthetic cream applied (venepuncture occurred some 80 min later by which time the anaesthetic cream had enabled the procedure to be painless). At age 15, in contrast, BP was taken while the individual was fasting and immediately before a (fasting) blood sample was taken. At age 17, the measurements were taken during the anthropometry assessments. At each age, two readings of systolic and diastolic BP and pulse rates were recorded and the mean of each calculated (for further details on methodology, see Brion *et al*).^23^

### Potential confounders

In this study, we allowed for a variety of social factors: (1) a family adversity score derived from 38 factors present in pregnancy (including maternal depression and anxiety) used as a continuous scale; (2) housing tenure (public housing vs rest); (3) household crowding (no. of persons in household/no. of rooms available); (4) stressful life events in the first half of pregnancy (sum of 44 possible events—treated as continuous scale); (5) smoking at 18 weeks gestation (yes vs no); (6) alcohol consumption at 18 weeks (yes vs no); (7) maternal age at birth; (8) parity (no. of previous deliveries); (9) maternal education level achieved; and (10) whether the child was breast fed. Since BPs varied with the sex of the child, this factor was also taken into account. We did not allow for birth weight or gestation as we considered these to be possibly on a common pathway from the exposure to the outcome.

### Statistical analyses

The statistical analyses first assessed the unadjusted associations between prenatal mercury and each of the BP and heart rate measures on continuous scales using multiple regression. Second, the analyses were adjusted for: sex, as well as the maternal and social factors as described above (model A). Finally, we incorporated selenium into the analyses by adding it as a covariate (model B). The models were then repeated for children whose mothers ate fish during pregnancy, and those who did not. We also examined the regression analyses to assess whether there were any interactions with sex.

Since these analyses were undertaken to assess possible adverse effects of mercury exposure, we designed the study to avoid type II statistical errors; consequently, we made no allowance for multiple testing, and considered all relationships with p<0.10.

## Results

Comparison of the outcome measures for the children for whom there were measures of maternal mercury were very similar to those of the whole tested population of ALSPAC (see online supplementary table S1), with the mean BPs of this study sample marginally lower (by about 0.5 mm Hg) and mean heart rates almost identical. The data used in these analyses (table 1) demonstrate that with increasing age, the mean systolic BP increased fairly steadily but with a sudden increase at age 15 and fall at 17. The mean diastolic pressure showed a similar pattern, but it was less dramatic. The resting heart rate, in contrast, fell steadily from age 7, but with an unexpected increase at age 15, and then a fall at age 17. It should be noted that at the 15-year assessment, the child's BP was measured when he/she was about to have a fasting blood sample taken—thus, the measurement is likely to have been influenced by his/her fasting state and by the fact that preparations for immediate venepuncture were being taken at the same time.

**Table 1:** Mean blood pressure and pulse levels (95% CIs) for the offspring for whom there was a prenatal blood mercury measure

Age at measurement (years)	N	Systolic BP*	Diastolic BP*	Pulse rate†
7	2207–9	98.43 (98.05 to 98.82)	56.09 (55.82 to 56.37)	83.19 (82.74 to 83.63)
9	2125	102.08 (101.69 to 102.46)	57.10 (56.84 to 57.37)	78.98 (78.54 to 79.42)
11	1950	104.98 (104.54 to 105.42)	58.50 (58.21 to 58.79)	75.54 (75.04 to 76.04)
13	1540	107.56 (107.05 to 108.07)	58.11 (57.79 to 58.43)	70.53 (69.97 to 71.08)
15	1494–5	122.97 (122.32 to 123.62)	66.47 (65.99 to 66.95)	74.23 (73.47 to 74.99)
17	1268	118.05 (117.44 to 118.66)	63.85 (63.49 to 64.21)	64.99 (64.46 to 65.53)

*mm Hg.

†BPM.

10.1136/bmjopen-2016-012425.supp1 supplementary tables

### Relationships between prenatal fish eating and mercury levels

The way in which the total blood mercury varies with the amount of fish eaten is shown in table 2. The means and medians are shown. Since the variation of mercury levels tends to be skewed,^15^ comparison of the medians is likely to be more meaningful. This shows that for white and oily fish, the biggest contrasts are between the women who do not eat fish and those who eat at least once in 2 weeks; in comparison, the levels of mercury show little variation with the frequency of fish consumption, particularly for white fish. For the subsequent analyses, we therefore define fish eaters as those who eat any type of white or oily fish at least once in 2 weeks.

**Table 2:** Mean (SD) and median levels of prenatal total blood mercury according to fish intake

Frequency	Oily fish			White fish		
	N	Mean (SD)	Median	N	Mean (SD)	Median
Never/rarely	1479	1.75 (0.94)	1.55	644	1.63 (1.02)	1.39
Once in 2 weeks	1139	2.28 (1.08)	2.08	1396	2.09 (0.99)	1.92
1–3 times/week	803	2.52 (1.16)	2.27	1374	2.35 (1.14)	2.10
4+ times/week	42	2.38 (1.02)	2.36	49	2.34 (1.15)	2.08
R^2^		8.13%			5.15%	

### Relationships between prenatal fish eating and offspring outcomes

The unadjusted mean BPs and heart rates of the offspring are compared between the children of women who ate fish in pregnancy and those who did not in table 3. Those children born to women who ate fish in pregnancy differed from those who did not in showing a significant reduction in systolic pressure at age 9, but a significant increase at age 11. There were no such differences in the mean diastolic BPs between the two groups, but the heart rates in the younger age groups were lower among the offspring of the fish eaters.

**Table 3:** Unadjusted mean (95% confidence limits) of offspring resting blood pressures and heart rates in childhood and adolescence according to whether the mother ate fish during pregnancy

Age of offspring	Systolic BP*		Diastolic BP*		Heart rate†	
	Mother ate fish		Mother ate fish		Mother ate fish	
Offspring (years)	Yes	No	Yes	No	Yes	No
7	98.4 (98.0 to 98.8)	98.7 (97.6 to 99.9)	56.0 (55.7 to 56.3)	56.4 (55.6 to 57.2)	83.0 (82.5 to 83.5)	84.3‡ (83.0 to 85.7)
9	101.9 (101.4 to 102.3)	103.0‡ (102.0 to 104.1)	57.0 (56.7 to 57.3)	57.8 (57.0 to 58.5)	78.8 (78.3 to 79.3)	80.5‡ (79.1 to 81.8)
11	105.1 (104.7 to 105.6)	103.7‡ (102.4 to 105.1)	58.5 (58.2 to 58.8)	58.2 (57.4 to 59.1)	75.3 (74.7 to 75.8)	77.0‡ (75.7 to 78.4)
13	107.3 (106.8 to 107.9)	108.7 (107.1 to 110.3)	58.0 (57.6 to 58.3)	58.6 (57.6 to 59.6)	70.4 (69.8 to 71.0)	71.6 (69.9 to 73.4)
15	123.1 (122.4 to 123.8)	122.2 (120.4 to 123.9)	66.7 (66.1 to 67.2)	65.7 (64.3 to 67.1)	74.0 (73.2 to 74.9)	75.7 (73.5 to 77.8)
17	118.0 (117.3 to 118.6)	119.3 (117.4 to 121.2)	63.8 (63.4 to 64.1)	64.5 (63.2 to 65.8)	65.0 (64.4 to 65.6)	64.8 (63.3 to 66.2)

Numbers in each cell can be obtained from online supplementary table S2.

*mm Hg.

†BPM.

‡Difference between fish eaters and non-fish eaters p<0.05.

### Relationships between prenatal mercury and offspring outcomes

Since the women who ate fish in pregnancy had higher blood mercury levels, but no evidence that their children's BPs differed from those whose mothers ate no fish, we anticipated that there would be little in the way of a consistent relationship between maternal blood mercury and offspring BP, but since the mean heart rates of the offspring of the fish eaters were lower, we predicted a possible negative association with heart rate.

The unadjusted and adjusted associations between mercury and systolic BPs are shown in table 4 for (1) all children, (2) those whose mothers ate fish and (3) those whose mothers ate no fish. Results of the analyses adjusted using model A show that for the whole sample, the associations between systolic BP and maternal mercury were positive for five of the six ages, but none approached statistical significance. Separate analysis of the children born to the mothers who ate fish showed similar positive regression coefficients at all ages, but not approaching statistical significance. However, for the children born to women who ate no fish, even though the CIs were wide, there was a significant association at age 11: β=+2.71(95% CI +0.77 to +4.66) mm Hg increase for each SD in maternal prenatal blood mercury (p=0.006). Comparison of the regression coefficients between the fish and non-fish-eaters revealed significant interactions at ages 11 and 15—the regression coefficient at 11 being significantly greater for the non-fish-eaters, but at 15, the coefficient for the fish eaters was greater than that for the non-fish-eaters.

**Table 4:** Relationship between prenatal mercury exposure and offspring systolic blood pressure (mm  Hg)

	All children		Mother ate fish		Mother ate no fish	
Age at measurement	N	β (95% CI)	N	β (95% CI)	N	β (95% CI)
7 years						
Unadjusted	2209	−0.13 (−0.48 to +0.22)	1825	−0.04 (−0.41 to +0.34)	270	−0.07 (−1.60 to +1.45)
		(p=0.465)		(p=0.854)		(p=0.926)
Model A	1874	+0.13 (−0.26 to +0.53)	1634	+0.16 (−0.26 to +0.57)	235	+0.16 (−1.53 to +1.85)
		(p=0.506)		(p=0.462)		(p=0.852)
Model B	1874	+0.10 (−0.31 to +0.51)	1634	+0.15 (−0.29 to +0.58)	235	+0.12 (−1.58 to +1.81)
		(p=0.625)		(p=0.511)		(p=0.894)
9 years						
Unadjusted	2125	−0.02 (−0.37 to +0.33)	1753	+0.23 (−0.16 to +0.62)	258	−0.60 (−2.05 to +0.86)
		(p=0.898)		(p=0.243)		(p=0.420)
Model A	1800	+0.18 (−0.22 to +0.58)	1581	+0.26 (−0.17 to +0.68)	213	−0.17 (−1.76 to +1.43)
		(p=0.377)		(p=0.240)		(p=0.837)
Model B	1800	+0.07 (−0.34 to +0.49)	1581	+0.19 (−0.26 to +0.63)	213	−0.27 (−1.85 to +1.32)
		0.734		(p=0.412)		(p=0.741)
11 years						
Unadjusted	1950	+0.11 (−0.30 to +0.51)	1621	−0.07 (−0.51 to +0.37)	223	**+2.14 (+0.39 to +3.89)**
		(p=0.606)		(p=0.756)		**(p=0.017)**
Model A	1658	+0.29 (−0.17 to +0.75)	1470	+0.02 (−0.47 to +0.50)	182	**+2.71 (+0.77 to +4.66)**
		(p=0.220)		(p=0.944)		**(p=0.006)**
Model B	1658	+0.21 (−0.26 to +0.69)	1470	−0.05 (−0.56 to +0.45)	182	**+2.64 (+0.70 to +4.58)**
		(p=0.377)		(p=0.842)		**(p=0.008)**
13 years						
Unadjusted	1540	−0.09 (−0.55 to +0.36)	1288	+0.01 (−0.49 to +0.51)	177	+0.42 (−1.77 to +2.60)
		(p=0.685)		(p=0.976)		(p=0.706)
Model A	1326	−0.03 (−0.55 to +0.49)	1171	+0.00 (−0.54 to +0.54)	151	+0.07 (−2.44 to +2.57)
		(p=0.916)		(p=0.994)		(p=0.958)
Model B	1326	−0.03 (−0.56 to +0.51)	1171	+0.02 (−0.54 to +0.58)	151	−0.00 (−2.54 to +2.53)
		(p=0.925)		(p=0.944)		(p=0.997)
15 years						
Unadjusted	1495	+0.17 (−0.41 to +0.74)	1242	+0.45 (−0.21 to +1.10)	177	−1.79 (−3.99 to +0.41)
		(p=0.573)		(p=0.181)		(p=0.110)
Model A	1283	+0.28 (−0.37 to +0.92)	1128	+0.41 (−0.28 to +1.10)	150	−**2.20 (**−**4.61 to +0.20)**
		(p=0.402)		(p=0.248)		**(p=0.072)**
Model B	1283	+0.19 (−0.48 to +0.85)	1128	+0.32 (−0.40 to +1.03)	150	−**2.21 (**−**4.64 to +0.21)**
		(p=0.586)		(p=0.384)		**(p=0.073)**
17 years						
Unadjusted	1268	−0.04 (−0.58 to +0.50)	1054	+0.03 (−0.57 to +0.63)	153	**+2.45 (+0.02 to +4.88)**
		(p=0.891)		(p=0.922)		**(p=0.049)**
Model A	1102	+0.12 (−0.44 to +0.69)	964	+0.16 (−0.43 to +0.75)	134	+1.74 (−0.82 to +4.30)
		(p=0.675)		(p=0.596)		(p=0.181)
Model B	1102	+0.15 (−0.44 to +0.73)	964	+0.19 (−0.42 to +0.80)	134	+1.73 (−0.86 to +4.31)
		(p=0.626)		(p=0.542)		(p=0.189)

Results are shown in bold typeface where p<0.10.

β gives change in BP for each SD of maternal blood.

Model A, adjustment for family adversity, housing tenure, household crowding, stress life events in first half of pregnancy, smoking midpregnancy, alcohol midpregnancy, maternal age, parity, maternal education, offspring breast fed.

Model B, model A+selenium level.

Similar analyses for the adjusted diastolic BPs are shown in online supplementary table S2 and summarised in table 5. Of the six measures for all children combined, there were four positively associated, three at the p<0.10 level. Considering the children of fish eaters separately, all the associations were positive, one being significant at the 0.05 level at age 9. For the children of the non-fish eaters, four were positive and two negative (one at the 0.10 level at age 15). The differences between the regression coefficients between the maternal fish and non-fish-consumers at age 15 were significant (p<0.05), the coefficient for the fish eaters being higher.

**Table 5:** Summary of adjusted associations (regression coefficients) between prenatal blood mercury and offspring diastolic blood pressures (for full details, see online supplementary table S2)

Age of offspring (years)	All cases	Mothers ate fish	Mothers ate no fish
7	+0.24 (−0.04 to +0.52)*	+0.26 (−0.04 to +0.55)*	+0.77 (−0.44 to +1.99)
9	+0.27 (−0.00 to +0.55)*	+0.35 (+0.05 to +0.64)**	−0.23 (−1.34 to +0.89)
11	+0.09 (−0.21 to +0.39)	+0.02 (−0.29 to +0.34)	+0.60 (−0.63 to +1.82)
13	+0.27 (−0.05 to +0.59)*	+0.28 (−0.06 to +0.61)	+0.51 (−0.93 to +1.96)
15†	−0.10 (−0.59 to +0.38)	+0.01 (−0.51 to +0.52)	−1.98 (−3.94 to −0.01)*
17	−0.01 (−0.38 to +0.36)	+0.06 (−0.32 to +0.43)	+0.18 (−1.78 to +2.14)

Regression coefficients give the increase (+) or decrease (−) in diastolic BP for each SD of mercury after controlling for model A.

*p<0.10, **p<0.05.

†Significant difference between regression coefficients offspring of fish and non-fish eaters at p<0.05.

Since we had shown differences in resting heart rates between children of fish and non-fish eaters, we had anticipated that there would be associations with maternal blood mercury levels. In fact, of the 18 adjusted associations we considered, none were significant at the 0.05 level and only 1 at the 0.10 level shown in online supplementary table S3 and summarised in table 6. There were no significant differences between the children of the fish and non-fish eaters.

**Table 6:** Summary of adjusted associations (regression coefficients) between prenatal blood mercury and offspring heart rate (for full details, see online supplementary table S3)

Age of offspring (years)	All cases	Mothers ate fish	Mothers ate no fish
7	−0.17 (−0.62 to +0.29)	−0.04 (−0.52 to +0.44)	−0.68 (−2.74 to +1.37)
9	+0.12 (−0.33 to +0.57)	+0.26 (−0.21 to +0.73)	+0.25 (−1.80 to +2.29)
11	−0.44 (−0.95 to +0.07)*	−0.27 (−0.81 to +0.28)	−0.32 (−2.35 to +1.70)
13	+0.04 (−0.52 to +0.59)	+0.15 (−0.44 to +0.73)	−0.19 (−2.84 to +2.46)
15	−0.15 (−0.90 to +0.70)	−0.04 (−0.86 to +0.78)	−0.56 (−3.46 to +2.48)
17	−0.08 (−0.62 to +0.46)	−0.10 (−0.68 to +0.49)	+0.72 (−1.34 to +2.79)

Regression coefficients give the increase (+) or decrease (−) in heart rate for each SD of mercury.

*p<0.10.

### Adjustment for selenium

In this study, blood levels of selenium are closely correlated with the blood levels of mercury in the study mothers (r=0.338; n=4134; p<0.001), and given that there is some evidence that blood selenium is positively associated with BP,^24^ we repeated the analyses allowing for selenium (model B in see online supplementary tables S1–S3). The results showed that the effect sizes for the relationships with mercury fell slightly in 78% of the systolic BP analyses, in 56% of those relating to diastolic BP, but only 11% of the analyses for heart rate. In comparison, the effect sizes increased between models A and B in 17%, 33% and 83%, respectively. In no instance did the change in effect size change the conclusions of the overall analyses.

### Gender interactions

We investigated the possibility that there might be differences in response to mercury between the genders. We therefore repeated all the adjusted analyses for all mother–offspring pairs allowing for a gender interaction. Of the 18 outcomes tested, there was only 1 with an interaction significant at the 0.05 level—systolic BP at 17 years of age (p<0.001). Further adjusted analysis showed that the relationship with prenatal mercury was positive for boys and negative for girls: boys β=+0.98 (95% CI +0.02 to +1.94); girls: β=−0.34 (95% CI −1.02 to +0.34). No other contrasts between the sexes showed similar variations (data not shown).

## Discussion

This set of analyses was devised to address the question as to whether exposure to mercury in pregnancy has a detectable effect on the resting BPs or heart rates of the offspring. In spite of deliberately not taking account of multiple testing so as not to fall into type II errors, we found few indications that in a normal population of fish consumers, there is an increase in BP in childhood and adolescence related to prenatal mercury level. Of the adjusted associations that were statistically significant at p<0.05, one showed a negative association with systolic BPs of the children aged 11 years of non-fish eaters (p=0.006) which remained significant after allowance for selenium (p=0.008), one showed a negative association with diastolic BP at age 15 (p=0.049) which was not formally significant after adjustment (p=0.055). In contrast, one set of analyses showed a positive association with diastolic BP of the 9-year-olds born to fish-eating women (p=0.021) but which was only marginally significant after adjustment for selenium (p=0.052). Thus, there was little evidence for any consistent relationships with prenatal mercury exposure.

The possible effects of prenatal mercury exposure and offspring BP were first assessed in a study in the Faroe Islands where the population mainly eat sea mammals rather than fish. The authors found that BP levels at age 7 showed an increase of 13.9 mm Hg diastolic and 14.6 mm Hg systolic as cord blood levels of mercury increased from 1 to 10 µg/L; increased mercury levels thereafter showed no further increase in BP.^8^ A later study examined the BPs at ages 12 and 15 in the Seychelles, a population eating a large amount of fish but not sea mammals. Prenatal mercury exposure was estimated from the mercury content of maternal hair collected postnatally. There was no association between prenatal mercury and offspring BP at age 12, but among boys at age 15, there was a positive association with diastolic BP after adjustment.^9^ The third study was from America (Project Viva in Massachusetts) where the authors found no association between prenatal mercury level and BPs at ages 3 or 7 years.^10^

Like the American study,^10^ we found no consistent relationship between prenatal mercury and offspring BP. It should be noted that the maternal blood mercury level in ALSPAC is low compared with that in the Faroes—ALSPAC median 1.86 µg/L and IQR 1.35 to 2.52 µg/L^15^ with only 0.4% >10 µg/L. Nevertheless, this distribution is somewhat greater than found among pregnant women in much of the developed world, including the USA.^25^ One other factor concerning the differences between the Faroes and Seychelles studies and our own concerns the statistical analyses. We controlled for a variety of socioeconomic variables as well as biological features such as maternal age, parity, smoking and alcohol intake as well as the sex of the child. We decided prior to the analyses not to allow for factors that may be on the biological pathway such as maternal hypertension and growth of the offspring with the intention to assess whether they were the mechanism by which any effect might have occurred. Since no consistent associations were shown, we did not investigate this pathway. In contrast, the adjustments in the Faroes study allowed for maternal hypertension, the child's weight and sex of the child; the Seychelles study adjusted for offspring birth weight, height and body mass index. Neither of these studies took account of any of the prenatal socioeconomic or biological factors included in our study.^8^ ^9^

### Strengths and weaknesses

This study benefits in: (1) having more participants studied than in the previous cohorts, and observations at six time points; (2) using data collected prospectively on potential confounders, with extensive information on prenatal lifestyle (cigarette smoking and alcohol ingestion), socioeconomic variation as well as biological features such as maternal age and parity, none of which appear to have been taken into account in two of the previous studies; (3) measures of BP and heart rate taken in similar standardised conditions at five of the six ages (the exception being at 15 years—see Methodology); (4) we were able to mirror the design of Vupputuri *et al*^5^ and compare the effects in offspring of fish eaters (who are likely to be associated with more methyl-mercury) compared with non-fish eaters (in whom the blood mercury is thought to be more likely to be predominately inorganic mercury). It should be noted that such analyses could not be performed in either of the other studies because almost the whole population ate some seafood. (5) We were able to assess whether blood selenium, levels of which are closely correlated with mercury, was blinding a relationships between mercury and the cardiovascular outcomes measured here.

The disadvantage of this study concerns the generally low levels of maternal prenatal blood mercury compared with that of the Faroes. Nevertheless, the positive trend of increasing systolic BP with prenatal mercury shown in the Faroes study applied only to the lower levels of blood mercury, and effects with offspring BP plateaued after cord blood levels of 10 µg/L.^8^ Consequently, our study should have had sufficient variation in blood mercury levels to be able to mirror the gradient at lower levels of mercury, should it exist.

## Conclusions

We have addressed the question as to whether maternal prenatal levels of blood mercury have any influence on the resting BPs or heart rates of the offspring from age 7 to 17. We were unable to detect any consistent robust associations. We conclude that for levels of mercury in a population of pregnant women who have a range of blood mercury levels of <10 µg/L, there is little to suggest an effect on the BP of the offspring during childhood and adolescence.
