# Supplementary material for: Are prenatal mercury levels associated with subsequent blood pressure in childhood and adolescence? The Avon prebirth cohort study
Source: BMJ Open. 2016 Oct 14;6(10):e012425. doi: 10.1136/bmjopen-2016-012425 (PMC5073590; doi:10.1136/bmjopen-2016-012425)
Supplement: supplementary tables [file bmjopen-2016-012425supp_tables.pdf]

Supplementary Table 1. Comparison between the mean blood pressures (BP) and heart rates between the whole ALSPAC cohort and the offspring for whom information is available on fetal exposure to mercury (study sample).

**Age of Offspring**	**SYSTOLIC BP (mmHg)**		**DIASTOLIC BP (mmHg)**		**HEART RATE[a]**	
	**ALL**	**Study sample**	**ALL**	**Study sample**	**ALL**	**Study sample**
7 years	99.0	98.4	56.5	56.1	83.1	83.2
9 years	102.8	102.1	57.5	57.1	79.0	79.0
11 years	105.6	105.0	58.8	58.5	75.7	75.5
13 years	107.6	107.6	58.2	58.1	70.6	70.5
15 years	123.6	123.0	67.8	66.5	74.2	74.2
17 years	118.5	118.1	63.9	63.9	64.8	65.0

[a]Beats per minute

Supplementary Table 2: Relationship between prenatal mercury exposure and offspring diastolic BP (mmHg). β gives change in BP for each standard deviation of maternal blood.

Age at Measurement	All children N	All children β [95% CI]	Mother ate Fish N	Mother ate Fish β [95% CI]	Mother ate no fish N	Mother ate no fish β [95% CI]
**7 years**						
Unadjusted	2207	+0.06 [-0.19,+0.31] (P=0.603)	1824	+0.12 [-0.16,+0.39] (P=0.404)	269	+0.89 [-0.19,+1.97] (P=0.106)
Model A	1872	**+0.24 [-0.04,+0.52] (P=0.099)**	1633	**+0.26 [-0.04,+0.55] (P=0.090)**	234	+0.77 [-0.44,+1.99] (P=0.211)
Model B	1872	**+0.25 [-0.04,+0.54] (P=0.096)**	1633	+0.26 [-0.05,+0.57] (P=0.106)	234	+0.80 [-0.42,+2.02] (P=0.197)
**9 years**						
Unadjusted	2125	+0.15 [-0.09,+0.39] (P=0.226)	1753	**+0.27 [+0.01,+0.54] (P=0.045)**	258	+0.04 [-0.97,+1.04] (P=0.945)
Model A	1800	**+0.27 [-0.00,+0.55] (P=0.054)**	1581	**+0.35 [+0.05,+0.64] (P=0.021)**	213	-0.23 [-1.34,+0.89] (P=0.690)
Model B	1800	+0.21 [-0.08,+0.49] (P=0.158)	1581	**+0.31 [-0.00,+0.61] (P=0.052)**	213	-0.29 [-1.39,+0.82] (P=0.610)

Supplementary Table 2 continued: Relationship between prenatal mercury exposure and offspring diastolic BP (mmHg). β gives change in BP for each standard deviation of maternal blood.

Age at Measurement	All children N	β [95% CI]	Mother ate Fish N	β [95% CI]	Mother ate no fish N	β [95% CI]
**11 years**						
Unadjusted	1950	+0.01 [-0.25,+0.27] (P=0.956)	1621	-0.04 [-0.33,+0.25] (P=0.789)	223	+0.76 [-0.31,+1.83] (P=0.164)
Model A	1658	+0.09 [-0.21,+0.39] (P=0.550)	1470	+0.02 [-0.29,+0.34] (P=0.890)	182	+0.60 [-0.63,+1.82] (P=0.337)
Model B	1658	+0.01 [-0.29,+0.32] (P=0.941)	1470	-0.06 [-0.39,+0.27] (P=0.714)	182	+0.07 [-1.91,+2.05] (P=0.943)
**13 years**						
Unadjusted	1540	+0.05 [-0.23,+0.33] (P=0.703)	1288	+0.17 [-0.14,+0.48] (P=0.277)	177	+0.69 [-0.63,+2.01] (P=0.301)
Model A	1326	**+0.27 [-0.05,+0.59] (P=0.098)**	1171	+0.28 [-0.06,+0.61] (P=0.103)	151	+0.51 [-0.93,+1.96] (P=0.483)
Model B	1326	**+0.30 [-0.03,+0.63] (P=0.076)**	1171	**+0.30 [-0.05,+0.65] (P=0.093)**	151	+0.61 [-0.85,+2.07] (P=0.411)

Supplementary Table 2 continued: Relationship between prenatal mercury exposure and offspring diastolic BP (mmHg). β gives change in BP for each standard deviation of maternal blood.

**Age at Measurement**	**All children N**	**β [95% CI]**	**Mother ate Fish N**	**β [95% CI]**	**Mother ate no fish N**	**β [95% CI]**
**15 years**						
Unadjusted	1495	+0.05 [-0.37,+0.48] (P=0.802)	1242	+0.15 [-0.32,+0.62] (P=0.538)	177	**-1.84 [-3.62,-0.06] (P=0.043)**
Model A	1283	-0.10 [-0.59,+0.38] (P=0.678)	1128	+0.01 [-0.51,+0.52] (P=0.978)	150	**-1.98 [-3.94,-0.01] (P=0.049)**
Model B	1283	-0.18 [-0.68,+0.32] (P=0.481)	1128	-0.10 [-0.63,+0.44] (P=0.726)	150	**-1.94 [-3.92,+0.04] (P=0.055)**
**17 years**						
Unadjusted	1268	-0.26 [-0.57,+0.06] (P=0.113)	1054	-0.20 [-0.54,+0.14] (P=0.256)	153	-0.17 [-1.87,+1.52] (P=0.839)
Model A	1102	-0.01 [-0.38,+0.36] (P=0.957)	964	+0.06 [-0.32,+0.43] (P=0.772)	134	+0.18 [-1.78,+2.14] (P=0.855)
Model B	1102	-0.03 [-0.41,+0.36] (P=0.899)	964	+0.06 [-0.32,+0.45] (P=0.744)	134	+0.07 [-1.91,+2.05] (P=0.943)

Model A = adjustment for family adversity, housing tenure, household crowding, stress life events in first half of pregnancy, smoking mid-pregnancy, alcohol mid-pregnancy, maternal age, parity, maternal education, offspring breast fed.

Model B = Model A + selenium level

Supplementary Table 3: Relationship between prenatal mercury exposure and offspring heart rate (beats/minute). β gives change in heart rate for each standard deviation of maternal blood.

Age at Measurement	All children N	All children β [95% CI]	Mother ate Fish N	Mother ate Fish β [95% CI]	Mother ate no fish N	Mother ate no fish β [95% CI]
**7 years**						
Unadjusted	2207	-0.27 [-0.67,+0.13] (P=0.183)	1824	-0.09 [-0.53,+0.35] (P=0.696)	270	-0.08 [-1.88,+1.72] (P=0.930)
Model A	1873	-0.17 [-0.62,+0.29] (P=0.477)	1633	-0.04 [-0.52,+0.44] (P=0.868)	235	-0.68 [-2.74,+1.37] (P=0.513)
Model B	1873	-0.04 [-0.52,+0.43] (P=0.857)	1633	+0.09 [-0.41,+0.59] (P=0.720)	235	-0.63 [-2.70,+1.43] (P=0.546)
**9 years**						
Unadjusted	2125	+0.05 [-0.35,+0.45] (P=0.796)	1753	+0.33 [-0.11,+0.77] (P=0.142)	258	+0.10 [-1.73,+1.93] (P=0.912)
Model A	1800	+0.12 [-0.33,+0.57] (P=0.600)	1581	+0.26 [-0.21,+0.73] (P=0.280)	213	+0.25 [-1.80,+2.29] (P=0.813)
Model B	1800	+0.16 [-0.31,+0.63] (P=0.501)	1581	+0.28 [-0.21,+0.77] (P=0.257)	213	+0.29 [-1.77,+2.34] (P=0.783)

Supplementary Table 3 continued: Relationship between prenatal mercury exposure and offspring heart rate (beats/minute). β gives change in heart rate for each standard deviation of maternal blood.

**Age at Measurement**	**All children N**	**β [95% CI]**	**Mother ate Fish N**	**β [95% CI]**	**Mother ate no fish N**	**β [95% CI]**
**11 years**						
Unadjusted	1950	**-0.44 [-0.89,+0.01] (P=0.057)**	1621	-0.25 [-0.74,+0.25] (P=0.335)	223	-0.29 [-2.06,+1.48] (P=0.748)
Model A	1658	**-0.44 [-0.95,+0.07] (P=0.089)**	1470	-0.27 [-0.81,+0.28] (P=0.338)	182	-0.32 [-2.35,+1.70] (P=0.754)
Model B	1658	**-0.45 [-0.98,+0.08] (P=0.094)**	1470	-0.30 [-0.86,+0.27] (P=0.304)	182	-0.26 [-2.28,+1.77] (P=0.804)
**13 years**						
Unadjusted	1540	-0.25 [-0.74,+0.24] (P=0.323)	1288	-0.25 [-0.74,+0.25] (P=0.335)	177	-0.68 [-3.02,+1.65] (P=0.564)
Model A	1326	+0.04 [-0.52,+0.59] (P=0.899)	1171	+0.15 [-0.44,+0.73] (P=0.624)	151	-0.19 [-2.84,+2.46] (P=0.888)
Model B	1326	+0.08 [-0.50,+0.66] (P=0.786)	1171	+0.16 [-0.45,+0.76] (P=0.611)	151	+0.00 [-2.67,+2.67] (P=0.998)

Supplementary Table 3 continued: Relationship between prenatal mercury exposure and offspring heart rate (beats/minute). β gives change in heart rate for each standard deviation of maternal blood.

**Age at Measurement**	**All children**		**Mother ate Fish**		**Mother ate no fish**	
	**N**	**β [95% CI]**	**N**	**β [95% CI]**	**N**	**β [95% CI]**
**15 years**						
Unadjusted	1494	-0.48 [-1.15,+0.19] (P=0.160)	1241	-0.21 [-0.97,+0.55] (P=0.588)	177	-1.11 [-3.84,+1.62] (P=0.422)
Model A	1282	-0.15 [-0.92,+0.62] (P=0.700)	1127	-0.04 [-0.86,+0.78] (P=0.919)	150	-0.56 [-3.46,+2.35] (P=0.706)
Model B	1282	-0.10 [-0.90,+0.70] (P=0.806)	1127	+0.00 [-0.85,+0.86] (P=0.995)	150	-0.44 [-3.36,+2.48] (P=0.766)
**17 years**						
Unadjusted	1268	-0.36 [-0.83,+0.12] (P=0.139)	1054	-0.37 [-0.90,+0.17] (P=0.177)	153	-0.09 [-1.94,+1.76] (P=0.920)
Model A	1102	-0.08 [-0.62,+0.46] (P=0.763)	964	-0.10 [-0.68,+0.49] (P=0.750)	134	+0.72 [-1.34,+2.79] (P=0.489)
Model B	1102	-0.08 [-0.64,+0.49] (P=0.792)	964	-0.09 [-0.69,+0.52] (P=0.778)	134	+0.75 [-1.34,+2.84] (P=0.481)

Model A = adjustment for family adversity, housing tenure, household crowding, stress life events in first half of pregnancy, smoking mid-pregnancy, alcohol mid-pregnancy, maternal age, parity, maternal education, offspring breast fed.

Model B = Model A + selenium level
